# Oxidative Stress and Mitochondrial Dysfunction in Cardiovascular Aging: Current Insights and Therapeutic Advances

**DOI:** 10.3390/biomedicines14010100

**Published:** 2026-01-03

**Authors:** Nabila Izzati Nur Azan, Norwahidah Abdul Karim, Nadiah Sulaiman, Min Hwei Ng, Asyraff Md Najib, Haniza Hassan, Ekram Alias

**Affiliations:** 1 Department of Biochemistry, Faculty of Medicine, Universiti Kebangsaan Malaysia, Cheras, Kuala Lumpur 56000, Malaysia; nabilaazan76@gmail.com (N.I.N.A.); norwahidah@ukm.edu.my (N.A.K.); 2 Department of Tissue Engineering and Regenerative Medicine, Faculty of Medicine, Universiti Kebangsaan Malaysia, Cheras, Kuala Lumpur 56000, Malaysia; nadiahsulaiman@ukm.edu.my (N.S.); angela@ukm.edu.my (M.H.N.); 3 Department of Pathology, Faculty of Medicine, Universiti Kebangsaan Malaysia, Cheras, Kuala Lumpur 56000, Malaysia; asyraff.najib@ukm.edu.my; 4 Department of Human Anatomy, Faculty of Medicine and Health Sciences, Universiti Putra Malaysia, Serdang 43400, Malaysia; nizahassan@upm.edu.my

**Keywords:** mitochondrial dysfunction, cardiovascular aging, oxidative stress, ROS, mitochondria dynamics, mitochondria-targeted antioxidants, mitochondrial transplantation

## Abstract

Mitochondrial dysfunction plays a central role in cardiac aging. Damaged mitochondria release excessive free radicals from the electron transport chain (ETC), leading to an increased production of reactive oxygen species (ROS). The accumulation of ROS, together with impaired ROS clearance mechanisms, results in oxidative stress, further disrupts mitochondrial dynamics, and diminishes bioenergetic capacity. Furthermore, the dysfunctional mitochondria exhibit an impaired endogenous antioxidant system, exacerbating this imbalance. These alterations drive the structural and functional deterioration of the aging heart, positioning mitochondria at the center of mechanisms underlying age-associated cardiovascular decline. In this review, we summarize the current evidence on how mitochondrial oxidative stress, mutations on mitochondrial DNA (mtDNA), and disruptions in the fission—fusion balance contribute to cardiomyocyte aging. This review also explores ways to mitigate oxidative stress, particularly with mitochondria-targeted antioxidants, and discusses the emerging potential of mitochondrial transplantation to replace dysfunctional mitochondria.

## 1. Introduction

A projection indicates that by the year 2050, individuals aged 60 years and older will account for nearly twice the current proportion of the global population [1]. Aging is defined as an evolving change in the dynamic between the promotive and protective mechanisms of aging [2]. In this context, a protective mechanism of aging is a biological process that limits age-related damage by repairing cellular injury or adapting metabolic function in response to environmental stress. In contrast, the promotive mechanism of aging is an age-dependent biological process whose effects become more noticeable as a person ages. These effects usually appear only in older individuals and may involve inherited traits that were passed on as they supported reproduction earlier in life [2]. From a biological perspective, aging can be defined as a persistent but normal climax of the loss of specific regenerative and bioprotective mechanisms that occur over time in an organism [3].

Aging is characterized by its hallmarks, which were first collectively defined in a 2013 review comprising nine defining characteristics of aging that are interconnecting [4]. The hallmarks were later reviewed, and three other hallmarks were added in 2023, making it the 12 hallmarks of aging [5]. Cumulatively, the hallmarks of aging are the loss of proteostasis, cellular senescence, stem cell exhaustion, genomic instability, telomere attrition, chronic inflammation, dysbiosis, altered intercellular communication, mitochondrial dysfunction, disabled macroautophagy, dysregulated nutrient sensing, and epigenetic alteration [5].

Aging induces inevitable changes throughout the body, initiating at the cellular level and extending to the largest organ—the skin—where aging shows its more apparent effect. Aging-related degenerative diseases span a broad spectrum across multiple physiological systems. With advances in disease prevention and treatment that have extended human life expectancy, aging remains as an inherent process that manifests its symptoms through the deterioration of the biology and physiology of an individual.

Aging is widely recognized as a primary risk factor for degenerative diseases, with mitochondrial dysfunction considered a central hallmark that contributes to the pathogenesis of aging-related diseases, such as neurodegenerative, cardiovascular, metabolic, musculoskeletal, sensory, and endocrine disorders [6]. Cardiac aging is physiologically characterized by progressive myocardial remodeling, left ventricular hypertrophy, and a gradual decline in both systolic and diastolic function [7].

Cardiomyocytes pose the most ubiquitous mitochondria compared to other organs and cells. Previous studies have shown that mitochondria supply over 90% of the adenosine triphosphate (ATP) source to the adult cardiomyocytes [8]. In addition, mitochondrial content occupies approximately one-third of the total cellular volume of adult cardiomyocytes [9,10,11]. These findings reflect the high energy dependency of cardiomyocytes, which rely on mitochondria for contractility, calcium handling, and cell survival.

**Figure 1:**
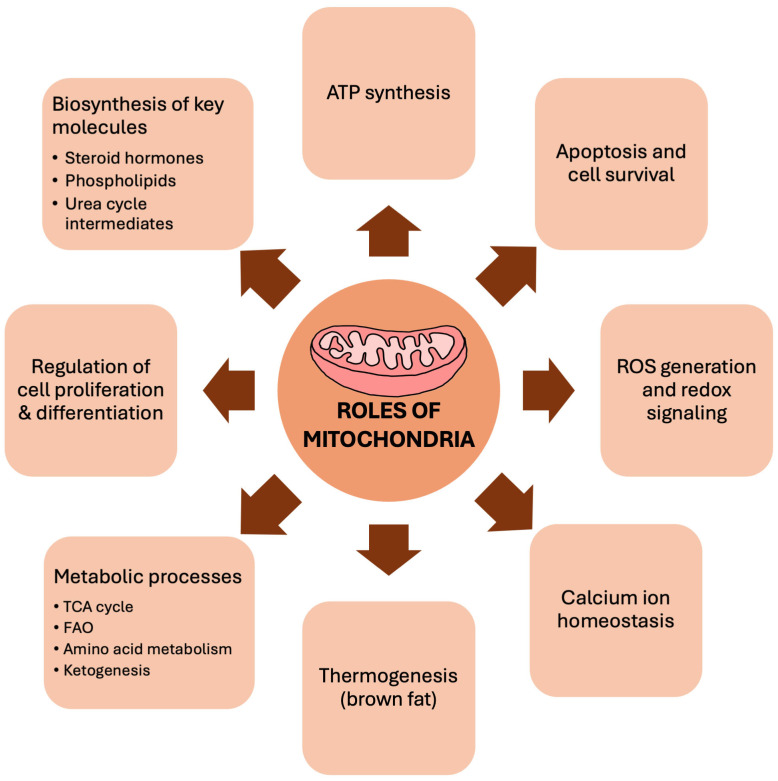
Roles of mitochondria. Mitochondria are more than the powerhouse of the cell. Beyond ATP generation, they function as central regulators of cellular homeostasis that contribute to cellular health, as illustrated above. ATP: Adenosine triphosphate; ROS: Reactive oxygen species; TCA cycle: Tricarboxylic acid cycle; FAO: Fatty acid oxidation.

Mitochondria are the principal site of energy production in the cell via oxidative phosphorylation (OXPHOS) through the ETC (see Figure 1 for more details on the other roles of mitochondria). With aging, the ability of mitochondria to store extra energy and supply them during stress declines, therefore increasing their vulnerability to stress and dysfunction. To maintain good cardiac health even with aging, proper regulation of mitochondrial homeostasis is crucial.

Previous studies that utilized animal models and human subjects have demonstrated that cardiac aging is intricately linked to alterations in mitochondrial morphology, increased mitochondrial ROS production, accumulation of mtDNA mutations, activation of the mitochondrial unfolded protein response, changes in NAD^+^ levels and sirtuin activity, and impaired mitophagy. Therefore, mitochondrial dysfunction is detrimental to the health and functionality of cardiomyocytes. Mitochondrial dysfunction has been considered an important hallmark for many diseases. With regards to mitochondrial dysfunction-related diseases, current therapies focus mainly on managing symptoms or downstream effects without addressing the root cause—to restore mitochondrial health and function. Since aging increases the cellular susceptibility to oxidative stress, results in mitochondrial dysfunction, and manifests as organ failure, mitochondrial-targeted therapies appear to be promising in promoting healthy cardiac aging.

This review aims to explore the fundamental role of ROS and oxidative stress in mitochondrial dysfunction associated with cardiac aging. Additionally, it highlights the potential of mitochondrial transplantation as a novel therapeutic strategy to restore mitochondrial health, enhance mitochondrial fusion–fission dynamics, and provide cardioprotection during aging.

## 2. Cardiovascular Aging

Cardiovascular aging refers to the progressive physiological, structural, and functional changes that occur in the heart’s vasculature over time [12,13]. Aging has been recognized as a major risk factor that increases susceptibility to cardiovascular diseases (CVDs) [12]. CVDs are the leading cause of death worldwide [12], which, with heart aging, can clinically manifest as coronary heart disease, peripheral artery disease, cerebrovascular disease, and atherosclerosis [13]. These age-related changes are accompanied by cellular maladaptive processes, including chronic inflammation, oxidative stress, and mitochondrial dysfunction [14].

### 2.1. Cellular Changes in Cardiovascular Aging

Aging induces structural changes in the heart. Cardiomyocytes isolated from aged (24-month-old) rats were shown to have damaged myofibrils with structural disruption on the sarcoplasmic reticulum tubule [15]. As the heart ages, concentric left ventricular hypertrophy often develops [16]. It is characterized by a thickening of the ventricular wall and a reduction in chamber size, thereby increasing the mass-to-volume ratio [17]. These structural changes seen in the aging heart result in systolic and diastolic performance decline [18]. Dysfunctions of the cardiac autonomic and conduction systems may also develop [16]. Aging also downregulates the expression of gene encoding for cardioprotective cytokine in epicardial adipose tissue [19].

The rate of cardiomyocyte death via autophagy and apoptosis are accelerated in aged hearts [20]. Such alterations cause the cardiac wall to thicken in an attempt to adapt and compensate for the shortfall in cardiac output, as well to accommodate the metabolic demand, despite an overall decline in cardiac efficiency [21]. Echocardiographic assessment revealed lower left ventricular ejection fraction, fractional shortening, and velocity of circumferential fiber shortening in 30-month-old rats compared to 4-month-old rats [18]. These differences indicate that aged rats have reduced resting systolic performance relative to young rats. Furthermore, aging also promotes fibroblast proliferation, increasing myocardial stiffness—a hallmark of maladaptive fibrosis—which impairs the mechanical ability of the left ventricle to pump blood efficiently [16].

Atrial changes are evident with aging, most notably in the form of left atrial dilation [16]. Older adults tend to exhibit greater left atrial enlargement than younger individuals. This dilation compensates for the elevated filling pressure caused by the increased stiffness and resistance of the thickened left ventricle [16]. An animal study demonstrated that aged hypertensive rats fed with high-fat diets had aggravated left atrial enlargement, which was associated with impaired left ventricular systolic function [22]. With aging, endocardial thickening becomes more pronounced, particularly in the left atrium and valves [23].

Diastolic evaluation demonstrated a prolonged isovolumic relaxation time and reduced tissue Doppler peak E velocities at both the septal and lateral mitral annulus, consistent with impaired early ventricular filling and delayed relaxation [18]. Collectively, these findings indicate modest but functionally relevant age-related declines in both systolic and diastolic performance, which, while compatible with adequate resting cardiac output, are likely to limit the capacity of the aging heart to meet increased hemodynamic demands.

**Figure 2:**
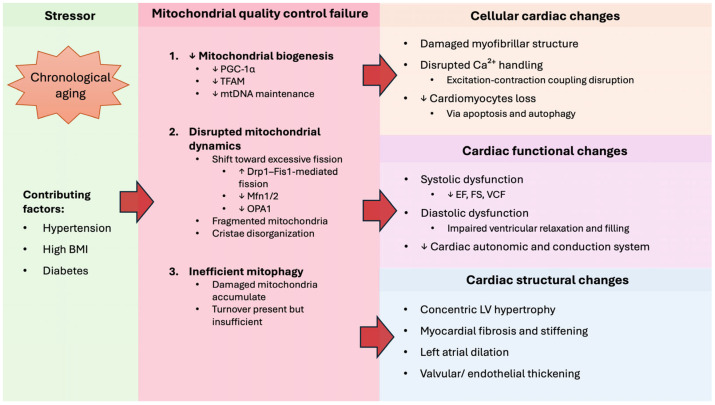
Aging-induced mitochondrial quality control failure as a unifying mechanism of cardiac aging that drives phenotypic changes within the heart at cellular, functional, and structural levels. This figure integrates the cellular and subcellular mechanisms described above, highlighting how mitochondrial dysfunction drives maladaptive remodeling in the aging heart. BMI: Body mass index; PGC-1α: peroxisome proliferator-activated receptor gamma coactivator 1-alpha; TFAM: mitochondrial transcriptional factor A; mtDNA: mitochondrial DNA; Drp1: Dynamin-related-protein 1; Fis1: Fission protein 1; Mfn1/2: Mitofusin 1/2; OPA1: Optic atrophy 1; EF: Ejection fraction; FS: fractional shortening; VCF: Velocity of circumferential fiber shortening; LV: Left ventricular. ↑ indicate “increase”, ↓ indicate “decrease”, whereas red-filled arrows mean “leads to”.

Other than aging, those structural changes of the heart could also be influenced by other contributing factors, which include hypertension, elevated body mass index, and diabetes [16]. These latter conditions act as cardiovascular risk factors that exacerbate hypertrophy. A study on old mice (24-month-old) confirmed that aging led to cardiac hypertrophy, as evident from a significant increase of the left ventricular myocardium [24]. In hypertension, the myocardium adapts by thickening the wall, which can worsen the condition [16]. Though gender has not been found to influence age-related enlargement of the left atrium [16], previous studies have shown that atrial fibrillation occurs more frequently in men, whereas women experience a higher incidence of thromboembolic events [25]. Conclusively, these structural and functional adaptations in the aging heart are intimately linked to mitochondrial dysfunction and progressive mitochondrial quality decline, as summarized in Figure 2.

### 2.2. Mitochondrial Dysfunction in Aging Heart

An aging heart exhibits altered cellular physiology that results in a decline in mitochondrial function and quality. Mitochondrial dysfunction is described as the inability of mitochondria—either acute or chronic—in maintaining normal mitochondrial metabolism and morphology [26]. Under physiological conditions, healthy mitochondria exhibit an elongated morphology that supports optimal ATP production and cellular homeostasis [15]. Poor mitochondrial function is reflected by a reduced oxygen consumption rate (OCR), reduced mitochondrial mass, reduced proteomic expression of ETC complexes, and elevated mitochondrial ROS production [27,28]. Damaged mitochondria pose altered characteristics that occur progressively until they become dysfunctional. Damaged mitochondria have fragmented and shorter mitochondria as they undergo more fission [15,29]. Furthermore, oxidative damage markers were detected higher in dysfunctional mitochondria, indicating that they have more mtDNA damage [29]. The higher intracellular calcium and sodium ions in isolated cardiomyocytes from aged rats (24-month-old) compared to those from young rats (6-month-old) could indicate more prolonged action potential [15].

While mitochondrial dysfunction can occur in any cell type, its occurrence and severity within the heart could worsen pre-existing cardiovascular disease progression within an individual [26]. There are several instances that mitochondrial dysfunction is linked with age-related cardiac problems. In aged mice (25-months-old), elevated mitochondrial oxidative DNA damage, evident by increased cardiac 8-hydroxy-2′-deoxyguanosine (8-OHdg), coincided with aggravated cardiac remodeling and an increased expression of cardiac senescence-related protein level [30], indicating that mitochondrial genomic damage is associated with structural and molecular hallmarks of cardiac aging. Ironically, aged mice (24-month-old) with cardiac hypertrophy showed no significant reduction in the number of mitochondria when compared to the young control (4-month-old) [24], suggesting that mitochondrial dysfunction in the aging heart is more of a qualitative than a quantitative impairment. In young mice, the increased mitochondrial oxidative stress caused by overexpression resulted in aortic stiffening and diastolic dysfunction, phenotypes typically observed in aged hearts [29]. Similarly, mice harboring a defective mtDNA proofreading polymerase γ (POLG) exhibited an accumulation of mtDNA mutations and developed premature cardiac hypertrophy [31], further demonstrating that mitochondrial genomic instability accelerates cardiac aging independently of chronological aging. These findings highlight that mitochondrial dysfunction can drive aging-like cardiovascular changes even with the absence of chronological aging. It is therefore clear that mitochondrial dysfunction is evident in aging-related cardiac models.

In the aging heart, mitochondrial biogenesis regulators, specifically mitochondrial transcriptional factor A (TFAM) and peroxisome proliferator-activated receptor gamma coactivator 1-alpha (PGC-1α), are altered [27,28]. Mitochondrial biogenesis refers to the cellular process of growing and dividing pre-existing mitochondria into new mitochondria [32]. In the study by Ungravi et al. (2008) [28], the messenger RNA (mRNA) expression of TFAM and PGC1-α in the aortic tissue were lower in old rats compared to young ones. Studies in C57BL/6 mice corroborate the age-related decline in the expression of mitochondrial biogenesis regulators TFAM and PGC-1α, accompanied by a progressive reduction in mtDNA copy number in aortic tissue from 8-, 22-, 44-, and 72-week-old mice; notably, mitochondrial metabolic activity, as measured by the oxygen consumption rate (OCR), exhibited a parallel age-graded decrease [27].

Jendrach et al. (2005) [33] reported that senescent primary human umbilical vein endothelial cells (HUVECs) contained swollen mitochondria with unstructured cristae, produced higher amounts of mitochondrial ROS, and harbored higher amounts of fragmented mtDNA. Similarly, in another study that compared the aortic vascular smooth muscle cells (VSMCs) of middle-aged mice (16-month-old) to young ones (4-month-old), higher ROS was reported in the middle-aged mice. Mitochondria isolated from the aortic VSMCs of 16-month-old mice exhibited higher levels of oxidized protein carbonyl residues, reduced ETC activity in Complexes I and III, and decreased OCR [34]. This might suggest that old age causes more oxidative protein damage and therefore impairs mitochondrial function. However, a study by Tyrrell et al. (2020) [35] that compared old (18–19 months) and young (2–3 months) C57BL/6 mice reported significantly higher OCR in the mitochondria of the aortic tissue of old mice. Further, there were more mitophagy-associated proteins and ubiquitinated mitochondria in the aortic tissue of the old mice, which might suggest that mitophagy is upregulated in the aortic tissue of aging organisms. Collectively, these findings suggest that mitochondrial dysfunction is not an isolated defect but a consequence of mitochondrial quality control disruption, with ROS-mediated oxidative stress acting as a key factor in this mechanism.

## 3. Oxidative Stress in Cardiac Aging

### 3.1. ROS-Mediated Oxidative Stress Results in Mitochondrial Dysfunction in Aging

The Free Radical Theory of Aging (FRTA) was first proposed by Denham Harman in 1956, suggesting that aging is a result of the accumulation of cellular constituent damage by free radicals with unpaired electrons [36]. Over time, this theory has evolved to encompass the broader term ROS, which includes both free radicals and non-radical molecules that contribute to oxidative stress [37].

Mitochondria are the primary source of ROS. ROS are produced as a natural by-product during OXPHOS in the ETC [38,39,40]. Electron leakage from the ETC may occur, particularly at Complexes I and III, and prematurely react with molecular oxygen. This process generates ROS such as superoxide (O_2_•^−^), hydrogen peroxide (H_2_O_2_), hydroxyl radicals (•OH), and singlet oxygen (^1^O_2_) [41]. Under normal physiological conditions, ROS play an important role as signaling molecules involved in cellular protection, the regulation of physiological processes, and the maintenance of cell survival within healthy limits [42]. ROS accumulate over time, and the prolonged failure to eliminate them results in oxidative stress [42].

Excess mitochondrial ROS contributes significantly to the protein remodeling observed in chronic heart failure [43]. The importance of oxidative stress as a central mechanism in the pathophysiology of cardiomyocyte senescence, hypertrophic remodeling, and the development of heart failure has been suggested in previous literature [44]. The imbalance between ROS production and neutralization induces oxidative damage that disrupts the cellular structure, proteins, and lipids, therefore inducing changes in DNA [42]. The accumulation of oxidative damage leads to the progressive loss of function in cardiac tissue [45].

**Figure 3:**
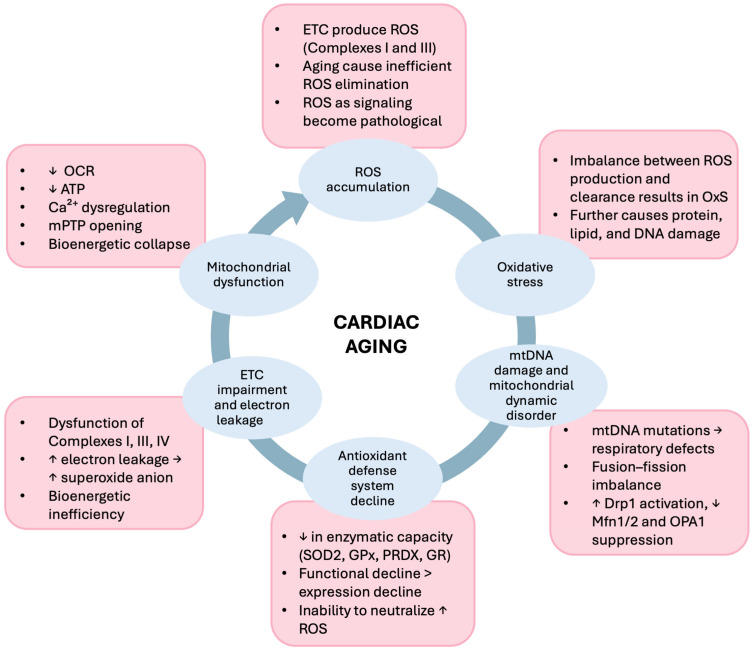
Self-perpetuating cycle of oxidative stress and mitochondrial dysfunction, in which excessive mitochondrial ROS acts as a primary driver that initiates and amplifies mitochondrial damage during cardiac aging. Aging-associated ROS accumulation disrupts mtDNA integrity, dynamics, antioxidant defense capacity, and respiratory chain efficiency, resulting in qualitative mitochondrial dysfunction that perpetuates oxidative stress and drives progressive cardiac aging. ETC: Electron transport chain; ROS: Reactive oxygen species; OxS: Oxidative stress; DNA: Deoxyribonucleic acid; mtDNA: mitochondrial DNA; Drp1: Dynamin-related protein 1; Mfn1/2: Mitofusin1/2; OPA1: Optic atrophy 1; SOD: Superoxide dismutase enzyme; GPx: Glutathione peroxidase; PRDX: Peroxiredoxins; GR: Glutathione reductase; ETC: Electron transport chain; OCR: Oxygen consumption rate; ATP: Adenosine triphosphate; mPTP: Mitochondrial permeability transition pore. ↑ indicate “increase”, ↓ indicate “decrease”, whereas → means “leads to”.

Experimental evidence indicates that ROS-mediated oxidative stress and mitochondrial dysfunction reinforce each other during cardiac aging. Age-related defects in mitochondrial ETC Complexes I, III, and IV increase electron leakage and superoxide production and hence further contribute towards the build-up of mitochondrial oxidative stress [46]. ROS-induced mtDNA damage also further impairs mitochondrial function, creating a vicious cycle of mitochondrial decline [25] (see Figure 3). Consistent with this mechanism, the accumulation of DNA mutations such as those induced by chronic oxidative stress leads to mitochondrial respiratory defects and premature cardiac aging. This is evident by the research on mice models with defective mtDNA polymerase who exhibited an accumulation of mtDNA mutations and premature aging phenotypes, including cardiomyopathy [31,38,47], characterized by cardiac hypertrophy, chamber dilatation, contractile dysfunction, and aggravated fibrosis [47].

The pathogenic interaction between ROS and mitochondrial dysfunction is also evident in genetic cardiomyopathies and may exacerbate cardiac aging. A previous study identified DES gene mutation as a pathogenic factor of genetic cardiomyopathy [48]. The DES gene encodes desmin, an intermediate filament protein that is essential for cytoskeletal integrity in muscle cells. The DES mutation impairs the anchoring, distribution, and structural integrity of mitochondria, resulting in mitochondrial dysfunction [48]. This secondary mitochondrial impairment in turn further amplifies mitochondrial ROS generation, culminating in progressive cellular dysfunction.

Mitochondria are highly dynamic organelles that constantly undergo fusion and fission in response to exogenous and endogenous triggers that directly influence their morphology [12,49]. Mitochondrial dynamics are regulated by key guanosine triphosphotase (GTPase) proteins that promote fusion—mitofusin 1/2 (Mfn1/2) and Optic Atrophy 1 (OPA1)—and proteins that promote fission—dynamin-related protein 1 (Drp1) and fission protein 1 (Fis1) [50]. Mitochondrial fusion involves the merging of individual mitochondria into elongated structures. Through this process, damaged and healthy mitochondrial content mix, diluting the localized damage and maintaining their mitochondrial function [12]. On the other hand, mitochondrial fission disintegrates individual mitochondria into fragmented pieces [12]. The removal of damaged mitochondria is performed via mitophagy [12]. The balance between mitochondrial fusion and fission is crucial in maintaining normal function. This mitochondrial dynamic is highly sensitive towards cellular changes and is susceptible to cell stress [44,51].

Impaired mitochondrial dynamics has become a major pathogenic mechanism underlying diseases associated with mitochondrial dysfunction [40,44]. The dynamic shift towards mitochondrial fission seen in aging exacerbates electron leakage from the ETC, elevates ROS production [41,52], and contributes to oxidative damage [29,41], eventually driving a self-perpetuating cycle of mitochondrial dysfunction [41]. The accumulation of ROS—and the resulting oxidative stress—has been recognized as the culprit that alters the expression and activity of mitochondrial dynamic-related protein.

Drp1 is sensitive to aging-associated signals [41]. Intracellular calcium homeostasis becomes dysregulated in aging [41]. The calcium dysregulation elevates cytosolic calcium levels in senescent cells, consequently activating calcium/calmodulin-dependent protein kinase (CaMK), which phosphorylates Drp1 at Serine616 residue, therefore promoting mitochondrial fission [52,53,54]. The phosphorylated Drp1 translocate to the mitochondria to mediate fission. This increase in mitochondria fission results in mitochondrial fragmentation, contributing to altered mitochondrial morphology observed in aged cells [15,29,41].

As the calcium dysregulation worsens and is compounded by sustained ROS accumulation, the pathological opening of the mitochondrial permeability transition pore (mPTP) is triggered [55,56]. In the aging heart, increased susceptibility to mPTP opening is closely linked to progressive mitochondrial dysfunction, which in turn accelerates the development of heart failure [57]. The mPTP opening leads to the pathological dissipation of the mitochondrial membrane potential (MMP), depletion of ATP, mitochondrial swelling, and the release of pro-apoptotic factors, ultimately resulting in cardiomyocyte cell death [55].

The energetic collapse resulting from mitochondrial dysfunction and mPTP opening has direct impacts on cardiomyocyte electrophysiology through the activation of ATP-sensitive potassium (K_ATP_) channels. These K_ATP_ channels, which serve as metabolic sensors that link cellular energy status to cardiac membrane excitability, open in response to reduced ATP availability [55]. In normal physiology, K_ATP_ channel activation imposes its cardioprotective effect by promoting hyperpolarization, therefore restricting calcium influx while reducing energy demand [55,58]. This mechanism prevents energy–supply mismatch, which prevents pro-apoptotic factor activation, mitigates mitochondrial dysfunction, and ultimately preserves heart cell viability under ischemic conditions [58]. However, this mechanism is disrupted in the aging heart [55,59]. Persistent oxidative stress, mitochondrial dysfunction, sustained ATP depletion, and redox imbalance in cardiac aging collectively promote the maladaptive signaling. Consequently, calcium homeostasis is disrupted, making the heart more susceptible to failure.

In addition, aging is also associated with a decline in AMP-activated protein kinase (AMPK) signaling, which normally suppresses DRP1 activity to prevent excessive mitochondrial fission [60]. This reduction further contributes to the enhanced mitochondrial fission observed in aging. In addition to regulating mitochondrial dynamics, AMPK acts as a central metabolic regulator that maintains cellular energy homeostasis by activating energy-generating catabolic pathways in response to changes in the AMP/ATP ratio [55]. AMPK also regulates mitochondrial biogenesis via the activation of the AMPK–SIRT1–PGC-1α axis and the MnSOD pathway, thereby increasing mitochondrial content while mitigating excessive ROS accumulation [55]. Elevated ROS levels can transiently activate AMPK signaling as a protective response to oxidative stress, enhancing autophagy and limiting oxidative damage [56]. However, under chronic oxidative stress conditions, this adaptive AMPK response becomes insufficient [61]. Aging-associated impairment of AMPK signaling further exacerbates mitochondrial quality control, resulting in mitochondrial dysfunction, progressive energy deficiency, cardiomyocyte dysfunction, and, ultimately, the development of heart failure [56,62].

Mfn1 and Mfn2 proteins, located on the outer mitochondrial membrane, are also susceptible to alterations in aging [12,61]. The high intracellular calcium levels observed in aging downregulate both Mfn1 and Mfn2 expression, thereby impairing mitochondrial fusion and shifting the balance toward fission in senescent cells [54].

OPA1 proteins, found localized in the inner mitochondrial membrane (IMM) [61], are expressed ubiquitously in the heart [62]. They play a central role in mitochondrial inner membrane fusion and the maintenance of cristae structure [12,61,62]. Mutations of OPA1 on highly conserved Arg781 and Arg824 disrupt the mitochondrial morphology in HeLa cells, which demonstrates the crucial role of OPA1 in maintaining the structural integrity of mitochondria [62]. A homozygous OPA1 deletion in mice was found to be embryonically lethal, while the heterozygous OPA1-knockout mice exhibited progressive cardiomyopathy characterized by a decrease in fractional shortening, a decrease in inotropy, and abnormal calcium transient [63]. These findings emphasize the importance of OPA1 for mitochondrial fusion, since even partial deletion could result in such detrimental effects.

OPA1-mediated mitochondrial membrane remodeling is dependent on its interaction with cardiolipin [62]. Cardiolipin enhances the GTPase activity of OPA1 [62]. Cardiolipin is also involved in the organization of ETC components and in apoptosis induction [64]. Cardiolipin levels have been shown to decline with aging [65]. This decline in aged cardiac tissue may impair the OPA1 activity and contribute to the progressive mitochondrial fragmentation observed during cardiac aging.

Experimental evidence demonstrates that the ROS-mediated alteration of mitochondrial dynamics occurs in cardiac aging. In a study in which mitochondrial dysfunction was induced in middle-aged mice through nicotinamide adenine dinucleotide phosphate oxidase 4 (NOX4) overexpression, the mice exhibited signs of decline in mitochondrial function in their left ventricular (LV) cardiomyocytes, as indicated by decreased OCR, reduced reserve respiratory capacity, and diminished activities of citrate synthase and Complex 1 compared to wild-type controls [29]. Those NOX4TG618 mice showed increased DRP1 phosphorylation at Ser616 residue and a decreased expression of MFN2, indicating an imbalance in mitochondrial dynamics favoring fission [29]. Observation through transmission electron microscopy (TEM) further confirmed the presence of small, fragmented mitochondria with an approximately threefold reduction in size relative to the control [29]. In contrast to the acute fission response observed under elevated ROS conditions, prolonged mitochondrial stress and persistent mtDNA damage in aging may trigger an alternative morphological adaptation. In the specific case of aged cells favoring fusion rather than fission in aging POLG mutator mice, TEM observation revealed the presence of enlarged mitochondria. The formation of these megamitochondria was hypothesized as an adaptive mechanism to dilute mtDNA damage to protect against ROS-mediated impairment [31].

Similarly, tissue comparison between 6 and 24-month-old rats indicated that cardiomyocytes isolated from the aged rats exhibited disrupted mitochondrial dynamics [15]. The accumulation of ROS observed in aged cardiomyocytes was associated with the decreased expression of Mfn2 and increased expression of Drp1, indicating reduced mitochondrial fusion and enhanced fission. Notably, treatment with antioxidants restored the balance of mitochondrial dynamics [15]. In aged (24-month-old) mice models, the mitochondrial dynamics are altered, as evidenced by the irregular 3D structure of interfibrillar mitochondria (IFM), poor mitochondrial organization, and inconsistent shape and size, as compared with young (4-month-old) mice [24]. To briefly summarize, the ROS-induced disruption of mitochondrial dynamics is responsible for driving mitochondrial dysfunction in cardiac aging.

### 3.2. Antioxidant System Declines in Aging

The body is equipped with a natural system consisting of antioxidant enzymes as a defense mechanism against ROS [66]. These defense systems include several crucial antioxidant enzymes, including superoxide dismutase 2 (SOD2), catalase, glutathione-dependent enzymes, and peroxiredoxins (PRDXs), which work synergistically with each other to neutralize the free radicals and prevent excessive ROS accumulation and oxidative damage [41,67,68,69]. The SOD2 enzyme neutralizes superoxide radicals (O_2_·^−^) by converting them into hydrogen peroxide (H_2_O_2_) and molecular oxygen (O_2_) [69]. The catalase (CAT) enzyme, mainly found in the peroxisome, mitigates the toxic effect of hydrogen peroxide (H_2_O_2_) by catalyzing its conversion into water and oxygen [70]. PRDX enzymes contribute to detoxifying H_2_O_2_ [67].

The glutathione system consists of several key antioxidant enzymes. They are crucial in reducing oxidized glutathione (GSSG) to reduced glutathione (GSH), which is important for neutralizing the H_2_O_2_. The normal physiological ratio of GSH to GSSG is 100:1 [71]. Glutathione peroxidase (GPx) enzymes have multiple functions. GPx1 reduces hydrogen peroxide and lipid hydroperoxides, thereby preventing excessive ROS accumulation [72], whereas GPx4 prevents lipid peroxidation [73]. Glutathione S-transferase (GST) protects the body against xenobiotics [74].

The accumulation of excessive ROS activates the endogenous antioxidant defense system [72]. However, the antioxidant enzyme levels decline and may be impaired with age [75]. The activity of the SOD-1 enzyme decreased significantly in healthy and hypertensive elderly human subjects [75]. Paradoxically, the same study found that the other antioxidant enzymes, including GPx-1, glutathione reductase (GR), and CAT, showed no significant relation to aging in both groups [75] despite the literature suggesting otherwise [76,77]. These discrepant findings are likely attributable to methodological limitations, particularly the small sample size (healthy elderly controls: 21 subjects; elderly hypertensive patients: 18 patients), which reduced the statistical power to detect moderate alterations in enzyme activity [75]. In addition, the lack of information on the nutritional status may have obscured age-related changes, given that nutritional status is a key determinant of antioxidant enzyme activity in elderly populations, thereby contributing to the observed discrepancies.

The importance of the antioxidant system in regulating ROS in cardiac tissues could be demonstrated by the fact that mice lacking manganese superoxide dismutase (MnSOD) developed cardiomyopathy [78]. Meanwhile, mice with CAT overexpression exhibit an extended lifespan and are protected against cardiac aging [79].

A more recent study reported that the activity of SOD2 and GPx antioxidant enzymes decreased significantly in old (15-month-old) and senescent (26-month-old) rat hearts, even though the SOD2 protein level was not different between the two groups [80]. Meanwhile, the activity and level of GR decrease significantly in both old groups [80]. An earlier study on aged rats found that the activity of SOD and CAT antioxidants was stable throughout their lives but markedly declined when the rats reached 24 months of age [81]. However, the study by Kaplan et al. (2019) [80] found no significant decrease in the CAT activity within the hearts of the old and senescent rats.

Kaplan et al. (2019) [80] also reported that the PRDX2 level was significantly reduced in senescent rats but unchanged in old rats. A recent study, which utilized an in vivo oxidative stress-induced model, indicated a lower level of PRDX3 in the mitochondria and PRDX4 in the cytosol of the myocardium of 24-month-old mice when compared to young ones [67]. The defects observed in the interfibrillar mitochondria (IFM) of the old mice [67] could suggest an association between mitochondrial dysfunction and the decline in antioxidant defense system and aging.

The findings of these studies demonstrate that the body’s antioxidant system declines with age. Other than aging, which impairs this antioxidant system, ongoing and excessive oxidative stress would also overwhelm the antioxidant defense system, hence further disrupting the balance between ROS production and clearance [41]. As endogenous antioxidant systems decline with age, supplementation with pharmacological antioxidants could become a strategy restoring the redox balance and preventing excessive ROS accumulation due to aging.

## 4. Mitochondrial-Targeted Therapy

### 4.1. Pharmacological Intervention

Given that mitochondria are the primary source of ROS, and antioxidants effectively neutralize them, mitochondria-targeted antioxidants may be particularly beneficial in the context of cardiac aging. Common examples of mitochondria-targeted antioxidants include, but are not limited to, Mitoquinone (MitoQ), SkQ1, and MitoTEMPO.

Mitoquinone (MitoQ) is a mitochondria-targeted derivative of coenzyme Q10 (CoQ10) [82]. CoQ10 is a natural molecule essential for the ETC with an antioxidant role, and its supplementation is beneficial to reduce the risk of CVD [83]. Nevertheless, CoQ10 has low bioavailability and uptake into mitochondria, which limits its clinical translation as a potent antioxidant [84]. To address this, MitoQ was formulated as a biochemically modified version of the natural antioxidant CoQ10, in which CoQ10 is covalently attached to a triphenyl phosphonium (TPP+) cation [85,86,87]. This modification enables selective accumulation of the specified molecule within the mitochondrial matrix, therefore enhancing its mitochondrial uptake [88].

MitoQ exerts its protective effects by directly targeting and mitigating oxidative processes that compromise mitochondrial integrity and function. Under oxidative stress, MitoQ reduces lipid peroxyl radical formation while suppressing lipid peroxidation, thereby preserving the mitochondrial structural stability [89]. MitoQ also act as a self-recycling antioxidant, enabling the sustained scavenging of ROS at low concentration [90]. Moreover, it protects the ETC, particularly the oxidation-sensitive Complex I, by preventing cardiolipin oxidation [90]. MitoQ further maintains mitochondrial network organization by preventing pathological fragmentation [91], suppressing oxidative stress-induced mPTP opening [90], and supporting mitochondrial quality control through enhanced mitophagy and restoration of biogenesis markers [82]. Collectively, these actions promote mitochondrial functional recovery, thereby enhancing cell survival.

Extensive preclinical studies have demonstrated the protective effects of MitoQ against cardiovascular aging seen in in vitro and animal models. Oral MitoQ supplementation ameliorated the age-related decline in endothelial dysfunction in old mice [92]. MitoQ supplementation to C57BL/6J mice with ascending aortic constriction appeared to provide protection against the pressure overload-induced cardiac remodeling and dysfunction [93]. MitoQ supplementation attenuated cardiac fibrosis, hypertrophic remodeling, and left ventricular dysfunction through the regulation of Nrf2, TGF-b1, NOX4, and long non-coding RNAs [93]. The intravenous administration of MitoQ prior to myocardial infarction induction significantly reduced mitochondrial ROS levels, and this suggests that MitoQ exerts cardioprotective effects by suppressing mitochondrial ROS and preventing its elevation in both wild-type and microRNA-210-deficient mice [94].

A recent study by Parker et al. (2025) [91] investigated the cardioprotective effects of MitoQ against acute and chronic oxidative stress in in vitro models. In human-induced pluripotent stem cell-derived cardiomyocytes (hiPSCM-CMs), MitoQ showed protection against acute oxidative stress by significantly blunting superoxide overproduction. When administered at the same dose under chronic oxidative stress conditions, MitoQ comparably reduced superoxide levels in hiPSCM-CM and H9C2 rat cardiomyoblasts (H9C2-rCM). In H9C2-rCM exposed to chronic oxidative stress, MitoQ treatment restored OCR, indicating preserved mitochondrial functional efficiency. In hiPSCM-CMs under chronic stress, MitoQ treatment attenuated mitochondrial hyperpolarization, thereby preventing premature electron leakage that could otherwise contribute to exercise ROS production [91].

An observation of isolated rat hearts with exposure to ischemia-reperfusion injury (IRI) showed that MitoQ administration via drinking water provides cardiac protection against loss of function, tissue damage, and mitochondrial damage [90]. This is supported by another study that also found that MitoQ supplementation provides a protective effect against IRI in mice [95]. Likewise, MitoQ administration in the heart of a mouse model showed decreased myocardial damage [96]. MitoQ also provides cardioprotective effects against heart failure, as demonstrated by its ability to rescue cardiac function in a mouse model of pressure overload-induced heart failure [97] and to attenuate cardiac remodeling by regulating the expression of genes associated with remodeling, thereby preventing heart failure in an ex vivo mouse model [93].

A clinical trial involving 6 weeks of MitoQ supplementation (20 mg/day dose) to middle-aged and elderly adults aged 60 to 79 years old, demonstrated the beneficial effects of oral MitoQ supplementation in vascular aging [88]. The increased level of plasma MitoQ not only decreased the plasma-oxidized low-density lipoprotein (oxidative stress marker) level by 13%, but it also led to improved endothelial function and reduced arterial stiffness [88]. The administration of 160mg MitoQ acutely improved vascular function [88].

Another well-studied mitochondrial-targeted antioxidant is SkQ1. The SkQ1 is a cationic plastoquinone derivative (10-(6′-plastoquinonyl) decyltriphenylphosphonium) formulated to target mitochondria and halt aging [98]. Similar to MitoQ, SkQ1 accumulates within the mitochondrial matrix, where it exerts its protective effects on mitochondrial function against oxidative stress [99]. SKQ1 prevents lipid peroxidation by forming a complex with cardiolipin [100], thereby limiting the cardiolipin availability as a substrate in fueling further oxidative damage [99]. SkQ1 also directly scavenges ROS while preserving the mitochondrial membrane integrity [99]. Following oxidation, SkQ1 can be reduced back to its active antioxidant form, SkQH_2_, enabling continuous redox cycling while rendering it a self-sustaining antioxidant similar to MitoQ [99]. The SkQ1 also prevents the downregulation of the cystic fibrosis transmembrane conductance regulator (CFTR), thereby preserving mitochondrial GSH levels and limiting H_2_O_2_ production [101], ultimately contributing to its antioxidant protective effects. Notably, an in vitro study from Antonenko et al. (2008) [99] suggests that SkQ1 exhibits greater antioxidant potency than MitoQ, reducing peroxyl radicals four-fold more efficiently.

While SKQ1 is widely known to be beneficial to the eyes (evident from the fact that it is an active compound that presents Visomintin, a dry eye medication) research exploring its effects on cardiac health has also been carried out [78,102,103]. Experimental evidence showed that SkQ1 provides a cardioprotective effect. An in vivo study revealed that lifelong treatment with SkQ1 demonstrated significant cardioprotective effects by reducing senile cardiomyopathy, cardiac hypertrophy, and fibrosis [78]. The SkQ1 treatment attenuated age-associated cardiac changes by approximately 50%, as reflected by preserved cardiac mass and reduced cardiac fibrosis. Moreover, SkQ1 reduced heart inflammatory response, as indicated by decreased leukocyte infiltration and decreased tissue inflammation [78].

The treatment of SkQ1 on high fructose-fed mice prevented cardiac hypertrophy as indicated by a lower left ventricular mass index and lower hypertrophy index [101]. The mRNA levels of cardiac hypertrophic markers, including atrial natriuretic peptide (ANP), brain natriuretic peptide (BNP), and beta-myosin heavy chain, were suppressed with SkQ1 treatment [101]. Moreover, SkQ1 ameliorated mitochondrial redox homeostasis by preventing the increase in the H_2_O_2_ production rate, preserving the GSH:GSSG ratio, preventing the decrease of ATP synthesis rates, and preserving maximal OCR [101]. In an in vivo study on rats with diabetic cardiomyopathy, which was induced with streptozotocin administration, treatment with SkQ1 showed reduced levels of free radical oxidation [104]. SkQ1 treatment restored aconitate hydratase activity, previously reduced by experimental hyperglycemia, indicating recovery of mitochondrial function from hyperglycemia-induced damage [104]. Co-treatment of SkQ1 and MitoQ on in vitro rat cardiomyoblasts with doxorubicin (DOX)-induced heart failure improved cell viability by reducing oxidative stress [103].

Another antioxidant drug used to target mitochondria is MitoTEMPO. This mitochondria-targeting antioxidant drug is a combination of antioxidant piperidine nitroxide TEMPO and the TPP cation [34]. With a large hydrophobic surface area, MitoTEMPO are able to penetrate the phospholipid bilayer easily and hence accumulate within the negatively charged mitochondrial matrix [34]. There was no evidence of toxic effects observed in MitoTEMPO-treated mice, as indicated by normal kidney and liver morphology and normal levels of alanine aminotransferase (ALT), creatine kinase (CK), and blood urea nitrogen (BUN) [34].

The findings from a previous study suggested that MitoTEMPO exhibited protective effects on cardiac health [15]. In the study, cardiomyocytes isolated from aged (24-month-old) Wistar male rats with a severe cardiac ultrastructural defect had more mitochondrial damage than the young (6-month-old) control [15]. MitoTEMPO acts as an SOD mimetic by mimicking natural antioxidant SOD to execute its function in reducing superoxide production [43,105]. Treatment with MitoTEMPO also restored mitochondrial membrane potential (MMP) by reversing membrane depolarization, which ensures normal energetic function in mitochondria [105]. In addition, MitoTEMPO treatment normalized mitochondrial dynamics, as shown by a significant decrease in Mfn2 expression and the increased expression of Drp1 [15].

In a guinea pig model of ischemic heart failure induced by a combination of ascending aortic constriction and beta-adrenergic stimulation, treatment with MitoTEMPO not only brought down the cellular ROS but, more impressively, could prevent and reverse heart failure by rescuing the contractile function of the heart [43]. All animals with aortic constriction survived after 3 weeks of treatment of MitoTEMPO with improved cardiac function [43].

The work by Vendrov et al. (2015) [34] is another study demonstrating the cardioprotective effects of MitoTEMPO by utilizing aged apolipoprotein E knockout (ApoE^−^/^−^) mice with aortic stiffening. MitoTEMPO treatment preserves aortic compliance, which improves vascular elasticity and reduces cardiac afterload, allowing the heart to pump blood more efficiently with smaller pressure changes. This change was attributed to the attenuation of fibrotic changes in the vascular wall [34]. Treatment with MitoTEMPO not only reduced both vascular and systemic ROS levels (as evidenced by the lower vascular and mitochondrial ROS, reduced vascular oxidative DNA damage marker 8-OHdG, and decreased plasma free 8-isoprostane, markers for systemic lipid peroxidation) but also abrogated mitochondrial protein oxidation and improved mitochondrial function [34].

Similar findings of attenuation in ROS production following MitoTEMPO treatment in an aging rat model were revealed in a later study [105]. Treatment with MitoTEMPO also appeared to increase Left Ventricular Developed Pressure (LVDP) amplitude, thereby preserving the depressed contractile activity and hence restoring cardiac function [105].

These mitochondria-targeted pharmacological interventions demonstrate cardioprotective effects mainly by attenuating oxidative stress and preserving mitochondrial function. However, their therapeutic efficacy remains limited, as they target the downstream consequences of oxidative damage rather than fixing the structural and functional mitochondrial deterioration. There has been increasing interest in alternative approaches that directly address mitochondrial dysfunction. Among these, mitochondrial transplantation has emerged as a promising strategy as it provides healthy, active, and viable mitochondria to the targeted cells [106].

### 4.2. Mitochondrial Transplant

The transfer of mitochondria from one cell to another is a natural biological phenomenon in multicellular organisms that can occur in physiological and pathological conditions [107]. Mitochondrial transfer was first shown to be possible by Spees et al. (2006) [108], in which they transferred mitochondria from hMSC and skin fibroblast cells into OXPHOS incompetent ρ^0^ cells via a co-culture method, thus restoring the function of mitochondria in producing ATP energy. Mitochondrial transfer describes the process of healthy donor cells transferring functional mitochondria to neighboring cells, thus restoring respiration, maintaining cellular homeostasis, and promoting cell survival [109]. The transfer occurs through multiple intercellular mechanisms [110].

Researchers have developed a mitochondrial transplantation method in which isolated exogenous mitochondria are transplanted into recipient cells to restore the mitochondrial function [111]. The aim is to increase the number of competent mitochondria while improving mitochondrial function within the cells [112]. The literature suggests that mitochondrial transplantation provides a cardioprotective effect [106,113,114]. Mitochondrial transplantation is a safe therapeutic option with demonstrated feasibility in clinical settings, as has been seen in a previous study wherein patients underwent an autologous intracardiac mitochondrial transplant without any of them developing any sign of abnormal heart rhythm afterwards [115].

Experimental evidence by Masuzawa et al. (2013) [114] indicates that mitochondrial transplantation provides a cardioprotection effect resulting from the enhancement of ATP production, proteome alteration, and cytokine induction. Using a rabbit model with ischemia-reperfusion injury (IRI), the mitochondrial transplantation carried out successfully restored the mitochondrial bioenergetic function, as evidenced by enhanced oxygen consumption and increased high-energy phosphate synthesis [114].

In a study in which mitochondria isolated from human cardiac fibroblasts were incorporated into human iPSC-derived cardiomyocytes, the results suggested that the integration of competent exogenous mitochondria into hosts could occur through fusion—as MFN1, MFN2, and OPA1 were observed to be localized at the fusion site—and might take part in facilitating the fusion of exogenous and endogenous mitochondria [116]. In addition, the study also found only a small amount of unphosphorylated DRP1, suggesting that no or little mitochondrial fission took place in the process [116].

Mitochondrial transplantation not only restores cellular metabolism but also provides a reparative response to the treated heart, promoting cardiac recovery. Masuzawa et al. (2013) [114] demonstrated that mitochondrial transplantation enhanced cell growth and tissue repair, as evidenced by the elevated levels of pro-repair signaling biomarkers, specifically EGF, GRO, and MCP-3. In addition, the zone with reperfusion injury showed decreased apoptosis [114]. In the rabbit IRI model treated with autologously derived mitochondrial transplant, the inflammatory biomarker (IL-6, TNF-α, and high-sensitivity C-reactive protein (hsCRP)) levels were either reduced or remained within the non-inflammatory range as measured by ELISA, suggesting that mitochondrial transplant does not elicit inflammatory response [114]. This is well-supported by the finding of the multiplex analysis on human peripheral blood mononuclear cells (PBMCs) that received xenogenic mitochondria from HeLa cells, in which no upregulation of inflammatory cytokines associated with rejection was demonstrated, coupled with the upregulation of cardioprotective cytokines [114].

Despite being largely experimental, mitochondrial transplantation has already been translated into human studies. Guariento et al. (2021) [115] reported a pilot study of mitochondrial transplantation in 10 young patients (<18 years old) with cardiogenic shock post-ischemia-reperfusion injury, and the results were compared to a control group of 14 patients that did not receive transplantation. The study demonstrated clear clinical benefits of mitochondrial transplantation, which was indicated by a higher rate of successful separation from post-cardiotomy extracorporeal membrane oxygenation (ECMO) and a reduced incidence of subsequent cardiovascular events [115].

Combined therapy of pharmacological intervention with mitochondrial transplant has also been incorporated. Using combined therapy of CoQ10 with mitochondrial transplant for treating IRI in aged rats, the treatment was revealed to have a better cardioprotective effect compared to the single intervention [117]. Further, reduction in infarct size and improved mitochondrial function were observed in the combined therapies [117]. Similarly, in another study, the combination of mitochondrial transplantation with Mitoquinone and melatonin treatment in aged rats was found to enhance myocardial function in the heart of aged rats with IRI more efficiently compared to single or dual therapies [118]. While these findings highlight the established usage of mitochondrial-targeting drugs, the potential usage of mitochondrial transplantation, and their synergistic usage, there are still challenges that need to be put into perspective.

### 4.3. Limitations and Future Direction

Generally, the literature suggests that both mitochondrial-targeting antioxidants and mitochondrial transplants are beneficial, but there are also caveats. The main limitation of pharmacological intervention is that there is no consistent dosage of antioxidant drugs between studies. The dosage of MitoQ differs in animal studies [85,86,92,119,120]. As for the applicability in humans, MitoQ is already being sold as a health supplement with a recommended dosage of 10 mg/day [121]. To date, there has been no published clinical trial recommending a dosage of SkQ1 and MitoTEMPO for cardiac health, and only a dosage related to eye treatment was documented for SkQ1 [98]. The existing evidence derived from animal models shows various dosages of SkQ1 [78,98,103]. Similar to MitoQ, the dosage of MitoTEMPO differs in animal studies [15,34,43,105,122,123].

Concern for these antioxidants’ target specificity toward damaged mitochondria has been raised in a previous study [124]. Essentially, the uptake of TPP+ cations depends on the mitochondrial membrane potential; therefore, hypothetically, damaged mitochondria may accumulate less drugs than the healthy ones. One study has provided experimental evidence on the poor effectiveness of SkQ1 and MitoTEMPO in providing their antioxidant effects [125].

Dose-related toxicity may occur in models treated with mitochondria-targeting antioxidant drugs. Pharmacological intervention—specifically MitoQ and SKQ1 at higher concentrations—can serve as pro-oxidants [126]. A different study revealed that at the dosage of 1 μM, there was evidence of the reduction and loss of the protective effect of SkQ1 and MitoQ, which further corroborates this claim [103]. Consistent with this finding, another study also found that SKQ1 can induce cell death at high concentrations [102].

Furthermore, certain pharmacological intervention requires sustained treatment; otherwise, the beneficial effect might not last. The beneficial effects of MitoTEMPO treatment on preventing and reversing established heart failure only lasted while the treatment was ongoing [43]. It was found that the withdrawal of MitoTEMPO treatment in the guinea pig model led to the recurrence of heart failure and reduced contractile function, though not to the extent of causing sudden cardiac death [43]. Some researchers took the approach of giving a supplementation of SkQ1 to their studied animal models throughout their lifespan, possibly due to the concern that the observed cardioprotective and anti-senescence effects of those mitochondrial targeted antioxidants might rely on the continuous presence of the antioxidants [43,78]. However, there is no evidence of a reverse beneficial effect of SkQ1 with discontinued treatment.

With limited studies conducted on humans for cardiac health, the long-term safety of antioxidants remains poorly characterized. The long-term consequences of their prolonged use remain unclear, with concerns of reduced drug responsiveness resulting in possible mitochondrial adaptation and the potential accumulation of drugs in non-targeted tissues. Additionally, their interactions with other pharmacological therapies for cardiovascular diseases have not been thoroughly investigated, leaving uncertainty about their safety profile in patients undergoing long-term treatment regimens.

Most experimental studies on mitochondrial transplantation in the heart have yielded promising results, with the primary focus on models of cardiac injury and disease. These findings provide important evidence of the feasibility of mitochondrial transplantation in restoring mitochondrial bioenergetics and improving cardiac function. However, translation into the context of cardiac aging remains limited. Notably, there are several recent studies that have begun to address this gap by incorporating mitochondrial transplant with pharmacological intervention in aged animal models [117,118]. To date, no mitochondrial transplantation study on aged human models has been conducted. The delivery of exogenous mitochondria specifically to the cardiac tissues remains a major obstacle to overcome. Another concern is the source of healthy mitochondria for mitochondrial transplant. In the current research setting, mitochondria are being isolated from sources that are xenogeneic, allogenic, and autologous (to avoid immunological response) to the recipient, as reviewed in previous literature [106]. While respiratory-competent mitochondria have been successfully isolated from skeletal muscle and liver and and cardiac tissue, these sources require surgical procedures to obtain [106], an approach that is clinically limiting due to its invasiveness, potentially higher complication risk, and the need for longer postoperative recovery.

Future research should be designed to address these limitations. Clinical studies on SkQ1 and MitoTEMPO are needed to elucidate their potential adverse effects and safety in humans. Long-term administration studies are essential to evaluate the safety and efficacy of these mitochondrial-targeted antioxidants. Further research involving both adult and aged human populations is also needed to determine the feasibility and therapeutic efficiency of mitochondrial transplantation across different physiological conditions. In addition, the development of minimally or non-invasive approaches for isolating and delivering healthy, respiratory-competent mitochondria could greatly enhance the applicability of this emerging therapeutic study. Ultimately, bridging the gap between promising preclinical findings and clinical translation requires a strategic approach to ensure a safe and effective treatment for cardiovascular diseases.

## 5. Conclusions

The accumulation of ROS ultimately results in mitochondrial dysfunction in the aging heart. The relationship between aging and mitochondrial dysfunction is bidirectional, forming a feedback loop in which mitochondrial dysfunction accelerates aging, while aging processes exacerbate mitochondrial health decline. The assault destructs the innate cellular physiology, structure, and functions of the heart, which becomes aggravated with aging. Current antioxidant therapies can offer cardioprotection effects but do not address the root problem of damaged mitochondria. Therapy with mitochondria-targeted antioxidants such as MitoQ, SkQ1, and MitoTEMPO is an under-studied option, with more research needed to address their limitations. Mitochondrial transplant offers an alternative way to provide healthy mitochondria to the targeted cell, thereby restoring mitochondrial function in producing ATP. With regards to aging, mitochondrial transplant, however, is still largely experimental, requires a more standardized method, and needs more translational studies involving humans. In conclusion, targeting mitochondria may be a promising approach in promoting healthy cardiac aging.

## Data Availability

The data presented in this study are available on request from the corresponding author due to data are not publicly available as they form part of ongoing or planned analyses.

## Abbreviations

The following abbreviations are used in this manuscript:

ETC	Electron transport chain
ROS	Reactive oxygen species
ATP	Adenosine triphosphate
OXPHOS	Oxidative phosphorylation
FAO	Fatty acid oxidation
NAD^+^	Nicotinamide adenine dinucleotide
CVD	Cardiovascular disease
OCR	Oxygen consumption rate
mtDNA	Mitochondrial DNA
DNA	Deoxyribonucleic acid
8-OHdg	8-hydroxy-2’-deoxyguanosine
POLG	Polymerase γ
TFAM	Mitochondrial transcriptional factor A
PGC-1 α	Peroxisome proliferator-activated receptor gamma coactivator 1-alpha
mRNA	Messenger RNA
HUVECs	Human umbilical vein endothelial cells
VSMCs	Vascular smooth muscle cells
FRTA	Free radical theory of aging
O_2_•^−^	Superoxide
H_2_O_2_	Hydrogen peroxide
•OH	Hydroxyl radicals
^1^O_2_	Singlet oxygen
DES	Desmin encoding protein
GTPase	Guanosine triphosphatase
Mfn1/2	Mitofusin 1/2
OPA1	Optic atrophy 1
Drp1	Dynamin-related protein 1
Fis1	Fission protein 1
mPTP	Mitochondrial permeability transition pore
CaMK	Calcium/calmodulin-dependent protein kinase
AMPK	AMP-activated protein kinase
SIRT1	Sirtuin 1
Arg781	Arginine at position 781
Arg824	Arginine at position 824
NOX4	Nicotinamide adenine dinucleotide phosphate oxidase 4
LV	Left ventricular
TEM	Transmission electron microscopy
IFM	Interfibrillar mitochondria
SOD2	Superoxide dismutase 2
PRDXs	Peroxiredoxins
CAT	Catalase
GSSG	Oxidized glutathione
GSH	Reduced glutathione
GPx	Glutathione peroxidase
GST	Glutathione S-transferase
GR	Glutathione reductase
MnSOD	Manganese superoxide dismutase
MitoQ	Mitoquinone
CoQ10	Coenzyme Q10
TPP+	Triphenyl phosphonium cation
Nrf2	Nuclear factor erythroid 2-related factor 2
TGF-b1	Transforming growth factor beta 1
RNAs	Ribonucleic acids
hiPSCM-CMs	Human-induced pluripotent stem cell-derived cardiomyoblasts
H9C2-rCMs	H9C2 rat cardiomyoblasts
IRI	Ischemia-reperfusion injury
SkQH_2_	Reduced (antioxidant) form of SkQ1
CFTR	Cystic fibrosis conductance transmembrane receptor
ANP	Atrial natriuretic peptide
BNP	Brain natriuretic peptide
DOX	Doxorubicin
ALT	Alanine aminotransferase
CK	Creatine kinase
BUN	Blood urea nitrogen
MMP	Mitochondrial membrane potential
K_ATP_	ATP-sensitive potassium channel
APoE^−^/^−^	Apolipoprotein knockout
LVDP	Left ventricular developed pressure
hMSC	Human mesenchymal stem cell
EGF	Epidermal growth factor
GRO	Growth-regulated oncogene
MCP-3	Monocyte chemoattractant protein-3
IL-6	Interleukin-6
TNF-α	Tumor necrosis factor-alpha
hsCRP	High-sensitivity c-reactive protein
ELISA	Enzyme-linked immunosorbent assay
PBMCs	Peripheral blood mononuclear cells
ECMO	Extracorporeal membrane oxygenation
